# No asymptomatic malaria parasitaemia found among 108 young children at one health facility in Dar es Salaam, Tanzania

**DOI:** 10.1186/1475-2875-12-417

**Published:** 2013-11-14

**Authors:** Gro EA Strøm, Marit G Tellevik, Maulidi Fataki, Nina Langeland, Bjørn Blomberg

**Affiliations:** 1 Department of Clinical Science, University of Bergen, Bergen, Norway; 2 National Centre for Tropical Infectious Diseases, Department of Medicine, Haukeland University Hospital, Bergen, Norway; 3 Muhimbili University of Health and Allied Sciences, Dar es Salaam, Tanzania; 4 Muhimbili National Hospital, Dar es Salaam, Tanzania

**Keywords:** Malaria, Asymptomatic infection, Dried blood spots

## Abstract

**Background:**

Asymptomatic malaria parasitaemia has been reported in areas with high malaria transmission. It may serve as a reservoir for continued transmission, and furthermore complicates diagnostics, as not all individuals with a positive malaria test are necessarily ill due to malaria, although they may present with malaria-like symptoms. Asymptomatic malaria increases with age as immunity to malaria gradually develops. As mortality and morbidity of malaria is higher among younger children it is important to know the prevalence of asymptomatic malaria parasitaemia in this population in order to interpret laboratory results for malaria correctly.

**Methods:**

A total of 108 children that had neither been treated for malaria nor had a fever the previous four weeks were recruited consecutively at a maternal and child health clinic (MCHC) in Dar es Salaam, Tanzania. A rapid diagnostic test (RDT) for malaria and dried blood spot (DBS) on filter paper were taken from each child. Social and clinical data were recorded. DNA was extracted from the DBS of study participants by a method using InstaGene™ matrix. PCR targeting the *Plasmodium* mitochondrial genome was performed on all samples.

**Results:**

Median age was 4.6 months (range 0.5-38). All the RDTs were negative. PCR was negative for all study subjects.

**Conclusion:**

The study suggests that asymptomatic malaria may not be present in apparently healthy children up to the age of three years in Dar es Salaam, Tanzania. However, because of the small sample size and low median age of the study population, the findings cannot be generalized. Larger studies, including higher age groups, need to be done to clarify whether asymptomatic malaria parasitaemia is present in the general population in the Dar es Salaam area.

## Background

In 2010, 655,000 people died of malaria, 91% of these in Africa, and there were approximately 216 million cases of malaria worldwide. Tanzania had approximately 5.48 million presumed and confirmed cases of malaria in 2011 [1].

Asymptomatic malaria parasitaemia is a known phenomenon in malaria-endemic areas with high year-round transmission [2,3]. It is indicative of partial immunity to malaria, with a low level of *Plasmodium* parasites, asexual or gametocytes, being detectable in the blood without the individual being ill. Infants are known to have a period of malaria immunity in the first one to two months of life, likely due to maternal antibodies, foetal haemoglobin and riboflavin deficiency, followed by an increased susceptibility to infection [4]. Studies have shown that in areas of intense transmission, the risk for severe disease is highest during the first two years of life [5]. Children frequently challenged with *Plasmodium* species acquire immunity resulting in fewer clinical infections of malaria and less severe presentation with increased age [5]. Infections with only gametocytes that for example persist post-treatment are always asymptomatic as gametocytes are non-pathogenic [6]. However, generally gametocytaemia accompanies asexual parasitaemia, with lower density in asymptomatic than in symptomatic infections [7], as the sexual parasites develop from asexual parasites [8]. Asymptomatic malaria parasitaemia in this study encompasses both asexual parasitaemia and gametocytaemia, as they are not differentiated by the methods used.

Asymptomatic malaria parasitaemia causes diagnostic challenges. In a clinical setting, a patient presenting with fever or other symptoms of malaria should always be treated for malaria when having a positive test result for malaria. However, a patient with asymptomatic malaria parasitaemia may present with a febrile illness with a different cause and with symptoms clinically indistinguishable from malaria, but requiring a different treatment despite a positive malaria result [9,10]. Clinicians must consider, investigate for and treat other causes of fever if malaria treatment fails or the child has other symptoms that are more likely attributable to other infections than malaria. The likelihood of a person with malaria parasitaemia presenting with symptoms and severity of disease correlates with the level of parasitaemia, although patients may be ill even with low parasitaemia [11]. Thus, level of parasitaemia is an insufficient indicator of whether malaria is the cause of a patient’s symptoms [12].

The prevalence of asymptomatic parasitaemia in areas with high malaria endemicity varies greatly among study locations. A study done in western Kenya identified a very high prevalence of asymptomatic asexual parasitaemia (73.8%) and gametocytaemia (33.8%) in children under five as assessed by thick blood film microscopy [13]. In Gabon, a study done in 2011 showed 1.7-8.7% asymptomatic parasitaemia in children when thick and thin blood films were examined [2]. The large variation may, to some degree, be attributable to the diagnostic methods used. Thick blood films, which are commonly used in many studies on asymptomatic parasitaemia [2,13,14], are known to be difficult to interpret [15].

Artemisinin combination therapy, which is currently recommended in Tanzania, has been shown to reduce the transmission potential of *Plasmodium* species compared to other common anti-malarials as they cause less persisting gametocytaemia post-treatment [16], although studies vary in their conclusions [17]. Gametocytes are necessary for the perpetuation of the life cycle of the *Plasmodium* species as they are ingested by mosquitoes and develop into sporozoites, which can be transmitted to new humans where they form the asexual stages that are pathogenic [18]. Patients with asymptomatic gametocytaemia, with or without concurrent asexual parasitaemia, may serve as reservoirs for the perpetuation of the malaria life cycle [19]. Treating asymptomatic gametocytaemia has been shown to have some value in reducing malaria transmission when combined with other interventions [20].

This study was performed to investigate for the presence of asymptomatic malaria parasitaemia among young children in Dar es Salaam, Tanzania.

## Methods

The study was performed as a collaboration between the University of Bergen and Muhimbili University of Health and Allied Sciences (MUHAS). Ethical clearance was obtained from the Regional Ethical Committee in Norway and MUHAS, and a research permit was obtained from Tanzania Commission for Science and Technology (COSTECH). Permission was also received from Magomeni Maternal and Child Health Clinic (MCHC).

At the Magomeni MCHC in Dar es Salaam Tanzania, 108 healthy children were recruited in May 2009. This is an area of high malaria transmission [21] with a prevalence of malaria among children six months to five years of age in 2011–2012, irrespective of symptoms, of 3.6% by RDT and 0.3% by thick blood film microscopy in the Dar es Salaam region [22]. The children were recruited consecutively from the population attending the clinic to weigh or vaccinate their child. Children were excluded from the study if they had been ill with fever or confirmed malaria, or received anti-malarials within the previous four weeks. Written, informed consent by signature or fingerprint was obtained from the parent or guardian of each child.

A simple questionnaire was completed for each child recording the child’s age, weight, gender, any recent travel (within the previous four weeks), whether the child slept under a mosquito net, the mother’s level of education and the number of siblings the child had.

An immunochromatography RDT cassette, ICT malaria Pf™ (ICT Diagnostics, Cape Town, South Africa), for *Plasmodium falciparum*, that detects histidine-rich protein 2 (HRP2) antigen in the blood, was performed on blood from a prick in the child’s finger according to the manufacturer’s instructions. The parent or guardian received the test results if they wished. RDT cassettes from the same lot were also tested on two children in a hospital ward with positive blood microscopy for malaria, to ensure that they were functioning correctly.

A drop of blood from the child’s finger was placed on a segment of a Whatman® Schleicher & Schuell filter paper, grade 589/2 (Whatman GmbH, Dassel, Germany). The dried blood spot (DBS) was stored in a sealed, airtight plastic pocket after air-drying completely. The DBS was later stored at-20°C until DNA extraction was performed.

### DNA extraction

For determining the limit of detection of the DNA extraction method, DBS were prepared from dilutions of a *P. falciparum-*positive sample with a parasitaemia of 20%, as identified by thin film microscopy, in *Plasmodium*-negative control blood. The dilutions ranged from 10^6^ parasites (p)/μl to 2 p/μl.

Harris Uni-Core™ puncher (Ted Pella, Inc, Redding, CA, USA) was used to punch out six pieces (or less, if less than six punches were available) of filter paper with dried blood, 3 mm in diameter. The filter paper punches were placed in a 1.5 ml Eppendorf tube. Between samples the puncher was cleaned by punching out six punches from a blank piece of filter paper and then plunging the tip of the puncher five times in sodium hypochlorite (NaClO) and 100% ethanol, respectively, followed by punching out three circles of blank filter paper. Using this cleaning procedure, no transfer of DNA between samples was detected.

DNA extraction was done by placing the punches in 200 μl InstaGene™ Matrix (Bio-Rad Laboratories, Hercules, CA, USA) and incubating at 56°C for 30 min, carefully vortexing after 15 min and after completed incubation. Another incubation for 8 min at 100°C was done, followed by centrifugation at 12,000 rpm for 2 min in an Eppendorf Centrifuge 5417C (Eppendorf AG, Hamburg, Germany). The supernatant was carefully removed and stored at -20°C until PCR was performed [23].

### PCR

PCR amplification of *Plasmodium* mitochondrial DNA, as described by Haanshuus *et al.*[24], but with a primer concentration of 1 μM, was performed on DNA extracted from the DBS. Analysis of PCR products was done by electrophoresis using 2% SeaKem™ agarose gel (Lonza, Rockland, ME, USA) with 1X GelRed™ (Biotium, Hayward, CA, USA).

### Statistical analysis

Basic statistics of general parameters was performed using IBM SPSSStatistics version 19 (SPSS Inc, IBM Company).

## Results

Demographics of the studied population are shown in Table 1. Sixty-seven children (62%) were under six months of age, 19 (17.6%) were six to 12 months, 15 (13.9%) were one to two years, and six (6.5%) were above two years. RDTs were negative for all the children.

**Table 1:** Demographics of study population (n = 108)

**Variable**	**N or Value (% or range)**
• Gender–female	63 (58.3)
• Age in months, median	4.6 (0.5-38.0)
• Weight in kg, mean	7.9 (3.5-15.4)
• Mosquito net used	105 (97.2)
• Mother with no or only primary school education	85 (78.7)
• Travel outside Dar es Salaam within the previous four weeks	14 (13.0)
• Used antibiotics within the previous four weeks	15 (14.0)
• Place of residence Dar es Salaam	103 (96.3)

With a positive PCR result for all triplicates of the 2 p/μl dilution, the InstaGene™ Matrix method could detect parasite concentrations of 2 p/μl or less. Mitochondrial PCR was negative for all study subjects.

## Discussion

No cases of asymptomatic malaria were identified in the population studied. This indicates that asymptomatic malaria parasitaemia is not common in children up to three years of age in the study area, and therefore that all positive malaria tests in this population should be considered true cases of clinical malaria. The results of the study may also indicate that the asymptomatic population studied does not act as a reservoir for perpetuating malaria transmission. However, age groups not studied here and populations in more rural areas may still have asymptomatic malaria gametocytaemia.

Recent studies find discrepancies in the sensitivities among different tests for malaria, with PCR generally detecting significantly more cases than microscopy and RDTs [25-27]. The PCR targeting mitochondrial DNA that was used in this study is very sensitive, detecting parasitaemia as low as 0.5 p/μl in DNA extracted from whole blood, and it was therefore not expected that any cases of asymptomatic malaria would be missed [24]. The level of detection of 2 p/μl or less for the InstaGene™ Matrix method for extraction of DNA from DBS is the same as has been found in a study done on extraction of blood from filter paper [28]. Not all the DBS were large enough for six punches, with 24% having less than five punches available. As a result suboptimal concentrations of DNA may have been obtained for some study subjects. The mitochondrial PCR is highly sensitive for gametocytes as these contain more mitochondrial organelles than asexual parasites [29] and it is therefore likely to be particularly sensitive in picking up low level gametocytaemia [24]. Asymptomatic malaria with persisting gametocytes is known to be frequent, for example, post-treatment [6,30]. A study done on the ICT malaria Pf RDT in Uganda showed high sensitivity (98%), specificity (72%) and negative predictive value (NPV) (98%) [31], also confirmed by a study by Singh *et al.*[32]. As the NPV and sensitivity are so high it is expected that the negative results are reliable. The ICT RDTs detect parasitaemia as low as approximately 300 p/μL, with high sensitivity (near 100%). Asymptomatic malaria infections generally have low parasitaemia, but threshold levels of parasitaemia vary from <100 p/μl to >1,000 p/μl depending on the intensity of transmission in the area and the age of those examined [33,34]. The RDT is therefore likely to miss some asymptomatic cases, but it would be expected that cases missed by the RDT would be identified by PCR. The consistency in results between PCR and RDT indicates that the negative results in this study are correct.

In a study conducted in the Dar es Salaam area in 2008 it was found that 83.2% of children under five had slept under a bed net the previous night and 30.9% had slept under an insecticide-treated net (ITN) [35]. In the current study the majority (97.2%) of the study participants were reported to be sleeping under a mosquito net. If this is correct, it may explain the lack of asymptomatic malaria parasitaemia in the children studied.

Children, for whom the parent or guardian reported febrile illness or anti-malarial treatment within the last four weeks were excluded from the study in order to reduce the risk of not detecting asymptomatic malaria infection due to recent anti-malarial use as well as to reduce the risk of detecting malaria parasitaemia due to symptomatic malaria illness. A study done by Hodel *et al.* in 2009 in Tanzania detected anti-malarials in the blood of over 80% of the recruited children that were under five years of age despite all reporting no anti-malarial use in the previous 28 days [36]. In the current study, 14% of the children had received antibiotics within the last four weeks. The type of antibiotic taken was unknown and could therefore be an antibiotic with anti-malarial properties. The finding of no asymptomatic parasitaemia in the study population may therefore, in some of the children included in the study, be due to recent treatment with antibiotics or with anti-malarials that was not reported.

The low median age of the study population, with 62% of participants less than six months of age, makes the findings less generalizable with respect to the general population in the area. It would be expected that younger children would not have as much asymptomatic parasitaemia as older children as it develops gradually during childhood when the child grows up in a malaria-endemic area with high perennial transmission [4,5]. In addition, the small sample size from only one health facility limits the generalizability of the findings to a broader population.

Despite the study’s limitations, the finding that asymptomatic malaria parasitaemia is rare suggest that positive malaria tests should always be interpreted as clinically relevant malaria in this population. The study also supports the notion that there is no asymptomatic parasite reservoir for transmission in young children, which encourages the continuation of current eradication efforts implementing early treatment with effective drugs and the use of ITNs and indoor residual spraying to eradicate malaria. The finding that PCR was negative for all the healthy children in this study lends support to the notion that false positivity is not a problem with the currently used mitochondrial PCR. A quantitative real-time PCR, targeting the same mitochondrial sequence, would be a valuable tool to further increase the understanding of low-level and asymptomatic parasitaemia and its impact on malaria transmission.

## Conclusion

This study indicates that there is likely no, or very little asymptomatic parasitaemia in very young children in the study area. All positive results for malaria in young children with clinically suspected malaria should be assumed to be illness due to malaria, and children with positive malaria test should receive adequate treatment for malaria. However, due to the small sample size and low median age of the population studied the results of the study cannot be generalized to other populations, health facilities or areas. A similar study should be performed on a larger population with a broader age range.

## Competing interests

The authors declare that they have no competing interests.

## Authors’ contributions

GEAS and BB were involved in all stages of the study. MGT was involved in the laboratory work especially with selecting methods for DNA extraction from dried blood spots. MF coordinated the fieldwork and arranged the cooperation with the MCHC. NL participated in the study design. All co-authors contributed to the data interpretation and writing of the manuscript. All authors have read and approved the final manuscript.
